# Supplementary material for: Satellite imagery reveals increasing volatility in human night-time activity
Source: Nature. 2026 Apr 8;652(8109):379–86. doi: 10.1038/s41586-026-10260-w (PMC13061621; doi:10.1038/s41586-026-10260-w)
Supplement: Supplementary file 1 — Supplementary Information [file 41586_2026_10260_MOESM1_ESM.pdf]

nature portfolio

https://doi.org/10.1038/s41586-026-10260-w

Supplementary information

Satellite imagery reveals increasing volatility in human night-time activity

In the format provided by the authors and unedited

Supplementary Sections:

1. Data Pre-processing
2. VZA-COLD Algorithm Implementation and Adaptations for Global Production

Supplementary Data Tables, supplied as separate files:

- Supplementary Table 1 | Ever-changed area experiencing at least one time ALAN change from 2014 to 2022 at global, continental, and country/territory levels.
- Supplementary Table 2 | Cumulative ALAN change area from 2014 to 2022 at global, continental, and country/territory levels by summing up the annual change areas of each year.
- Supplementary Table 3 | Cumulative ALAN radiance change for the net, brightening, and dimming changes from 2014 to 2022 at global, continental, and country/territory levels.
- Supplementary Table 4 | Averaged ALAN change intensity for the net, brightening, and dimming changes at global, continental, and country/territory levels.
- Supplementary Table 5 | Trends in annual ALAN radiance change, change area, and change intensity for the net, brightening, and dimming changes at country/territory levels.

Supplementary Data Figures:

- Supplementary Fig. 1 | Abrupt versus gradual ALAN change frequency for the top 100 changing countries/territories (2014-2022).
- Supplementary Fig. 2 | Country/territory level ALAN changes from 2014 to 2022.
- Supplementary Fig. 3 | Analyzed nighttime light area used for global ALAN change detection (70°N-60°S).
- Supplementary Fig. 4 | Illustration of VZA-COLD intermediate change detection progress.
- Supplementary Fig. 5 | Spatial distribution of the global validation sample set.

1. Data Pre-processing

Before applying the VZA-stratified COntinuous monitoring of Land Disturbance (VZA-COLD) change detection algorithm[1], two critical preprocessing steps were implemented on the daily VNP46A2 NTL data to enhance input time series quality and optimize computational efficiency.

The first step involved filtering low-quality daily images to mitigate sensor anomalies and residual environmental contamination. While the standard Black Marble Quality Assessment (QA) flags are effective in removing many pixels affected by clouds or snow[2,3], systematic sensor errors (such as those documented by NASA's Land Product and Data System, MODAPS, including "safe mode" events, "false fire" detections, or "day mode" incursions during nighttime orbits) or subtle cloud/aerosol contamination not fully captured by the QA flags can persist[4]. These issues can introduce spurious high radiance values, particularly noticeable in regions that do not usually have artificial light[5]. Such artifacts can lead to a significant number of false brightening change detections (commission errors), often appearing as salt-and-pepper noise or large anomalous patches in the final change maps. To address this systematically on a global scale, we developed and applied a time-series-based outlier detection method designed to identify and remove entire contaminated daily images at the VIIRS tile level. The rationale behind this approach is that regions with low NTL intensity (e.g., rural areas, naturally lit landscapes) generally exhibit much lower intrinsic temporal variability compared to brightly lit urban centers. Consequently, an anomalous increase in the average radiance of these typically stable dark pixels across an entire image is a strong indicator of widespread data quality issues within that specific image, often stemming from faulty instrument granules or unflagged atmospheric phenomena affecting large areas.

The procedure was as follows: for each VIIRS tile and each day from 2013 to 2023, we first identified all pixels with VNP46A2 radiance values below a defined low-light threshold of 10 $nW\cdot cm^{-2}\cdot sr^{-1}$. Then, we calculated the mean radiance of these identified low-light pixels for that specific image (tile-day). This resulted in a time series of daily low-light mean radiance values for the entire 2013-2023 period for each of the 333 VIIRS tiles. We then calculated the long-term mean and standard deviation of this low-light mean time series for each tile as shown in equation (1). Outlier images were identified by flagging any day with unusual high mean radiance of low-light regions (only for pixels < 10 $nW\ cm^{-2}\ sr^{-1}$) for a tile that fell outside the z-score value of 75% confidence interval (CI) of that tile's long-term low-light mean time series, as shown in equation (2). All observations from these flagged images (i.e., the entire tile for that specific day) were completely removed from the input time series dataset prior to any change detection analysis. This preprocessing step proved highly effective in filtering out images significantly affected by systematic sensor errors or widespread residual environmental contamination, thereby

substantially reducing the prevalence of spurious, salt-and-pepper ALAN change detections in the final product.

The second pre-processing step involved defining the “analyzed nighttime light area” to focus computational resources and the subsequent analysis on regions with a historical or current presence of artificial light. ALAN changes, by definition, can only occur in locations that have been illuminated at some point. Including pixels that remain consistently lit only by natural light throughout the entire study period in the change detection process is computationally inefficient and can also lead to an inflation of commission errors caused by instrument noise or changes in airglow around the DNB sensor's detection limit. To optimize the processing pipeline and improve the robustness of the change detection, we generated a nighttime light area mask. This mask was created by applying a minimum light threshold to the pre-processed NTL time series (after the removal of low-quality images). We used a conservative threshold of 1.0 $nW \cdot cm^{-2} \cdot sr^{-1}$, which is twice the nominal NTL detection limit ($L_{min} \approx 0.5$ $nW \cdot cm^{-2} \cdot sr^{-1}$) specified for the Black Marble product[2]. This provides a buffer against noise for very low light signals. To determine if a pixel met this threshold, we calculated a rolling median radiance using a window of 14 consecutive clear (cloud/snow-free) observations. The window size of 14 observations was chosen to align with the number of consecutive anomalous observations required to confirm an ALAN change within the VZA-COLD algorithm (see Supplementary Section 3 in Li et al.[1]), thereby ensuring consistency between the masking criteria and the change detection parameters. A pixel was classified as belonging to the analyzed nighttime light area if its rolling median nighttime light radiance exceeded the 1.0 $nW \cdot cm^{-2} \cdot sr^{-1}$ threshold at any point during the 2013-2023 observation period. All pixels that never met this condition were masked out and excluded from subsequent change detection analysis.

The final analyzed nighttime light area, after applying this mask, covered 15 million km^2, which represents 10% of the global terrestrial land surface within the defined 70°N-60°S latitude range (Supplementary Fig. 3, Extended Data Table 3). This focused approach ensures that computational efforts are concentrated on regions where human activity signals are most likely to be present and detectable. In some high-latitude portions of this analyzed area, frequent and bright auroral activity[6], if persistent enough to pass the rolling median filter, might contribute to a pixel being included in this initial mask. However, the VZA-COLD change detection algorithm itself, requiring 14 consecutive anomalous observations against a modeled seasonal baseline, is designed to be robust against transient natural phenomena like most auroral displays or wildfires, which typically do not exhibit such sustained, model-breaking behavior consistent with ALAN changes. We chose to retain these areas in the analyzed domain to avoid inadvertently excluding locations with actual ALAN signals, especially in regions with low but growing light activity. However, because some of these marginal pixels may never experience actual ALAN change, the estimated

rate of change is likely a conservative one, slightly underestimating the true proportion of areas experiencing ALAN dynamics.

$$\mu_{i,tile} = \frac{1}{N_{i,tile}} \sum_{p \in \mathcal{L}_{i,tile}} DNB_{i,tile} \qquad (1)$$

where,

$\mathcal{L}_{i,tile} = \{p \in tile_i \mid DNB_{i,tile} < \tau\}$ is the set of low-light pixels for the VIIRS geographic projection 10°×10° $tile$ on day i.

$N_{i,tile} = |\mathcal{L}_{i,tile}|$ is the number of low-light pixels for day i.

$\mu_{i,tile}$ is the daily mean DNB radiance of low-light pixels of the *tile* on day i.

$$\mu_{i,tile} < \overline{\mu_{tile}} - z * \sigma_{tile} \quad \text{or} \quad \mu_{i,tile} > \overline{\mu_{tile}} + z * \sigma_{tile} \qquad (2)$$

where,

$\overline{\mu_{tile}}$ is the averaged DNB radiance means for low-light pixels from 2013 to 2023.

z is the confidence threshold coefficient of 1.15 for approximately a 75% confidence interval assuming normality.

σ_{tile} is the standard deviation of the image based low-light means from 2013 to 2023.

2. VZA-COLD Algorithm Implementation and Adaptations for Global Production

The development, theoretical underpinnings, and initial validation of VZA-COLD for daily NTL data were comprehensively detailed in Li et al. (2022)[1]. VZA-COLD incorporates several key innovations tailored to the specific characteristics and challenges of daily VIIRS DNB NTL data at the pixel level. Supplementary Fig. 4 illustrates how VZA-COLD identifies nighttime light change resulting from new construction. Its approach involves mitigating the significant variability introduced by sensor viewing geometry by stratifying daily NTL observations for each pixel into four distinct VZA intervals: 0-20° (near-nadir), 20-40°, 40-60° (off-nadir), and a composite 0-60° (all available data). This stratification aligns with approaches used in generating higher-level Black Marble composite products[7]. For each of these VZA strata, a harmonic time series model described in equation (3) was continuously estimated using robust regression techniques[8]. This model captures both intra-annual seasonality (e.g., due to vegetation phenology affecting light obstruction or seasonal variations in human activity) and inter-annual trends (e.g., due to economic growth, urbanization, or policy changes). Supplementary Fig. 4a illustrates the successful initialization of harmonic time series models (solid red curves) using daily VIIRS DNB NTL observations. The change detection component of VZA-COLD continuously predicts future observations based on the current model parameters for each stratum. An observation was flagged as an anomaly if the residual (the difference between the observed and predicted NTL value) exceeds a dynamically calculated threshold (following the optimal parameter setups mentioned in Li et al., 2022[1]). This threshold was based on the model's Root Mean Square Error (RMSE) and a pre-defined change probability (set to 75%). A breakpoint, signifying an abrupt ALAN change, was confirmed if a sequence of 14 consecutive anomalous observations occurred in any of the VZA strata. Once an abrupt change was confirmed, the time series for all four VZA strata for that pixel were segmented at the identified breakpoint time (labeled as the start date of the confirming 14 consecutive anomalous dates) to ensure temporal consistency across the models. Supplementary Fig. 4b illustrates that the abrupt change (brightening) was identified by consecutively comparing the current model prediction (dashed red curve) with the actual observations (black dots) from the VZA interval 0-20°. Then, new harmonic time series models were initialized and then used to search for the next change until all observations were appended (Supplementary Fig. 4c). After segmentation by abrupt changes, each resulting time segment was then assessed for gradual ALAN change based on all NTL observations. A segment was identified as undergoing gradual change if the linear trend coefficient (slope term, b_i, in equation (3)) for that segment is statistically significantly different from zero ($p < 0.05$) (for example, the second segment of the composite 0-60° in Supplementary Fig. 4c).

For this global-scale application and production, three critical adaptations were made to the previously published VZA-COLD algorithm to address specific challenges of global data

processing and high-latitude environments. A global calibration dataset of 610 representative pixels, previously compiled from diverse geographic regions, land cover types, and latitudes for algorithm development[1], was applied to decide the optimal skipping fraction and moving window length, ensuring robust and consistent detection of ALAN changes while maintaining computational efficiency.

First, a dynamic harmonic model period was implemented specifically for high-latitude regions. The standard harmonic model used in VZA-COLD (and the original COLD[9]) assumes a fixed annual period ($T = 365.25$ days). This assumption is frequently violated in high-latitude areas due to extended periods of polar day (resulting in no nighttime observations) and persistent, extensive snow and ice cover (which can either mask the NTL signal or cause high albedo effects that contaminate observations[6]). Attempting to fit a fixed annual model to such incomplete annual data records leads to poor model fits and a substantial number of commission errors (i.e., misclassifying stable periods as change). To overcome this critical limitation, we developed and implemented a data-driven, pixel-specific dynamic period for the harmonic model. The procedure involved, for each pixel, analyzing the entire 11-year (2013-2023) time series of valid, pre-processed (cloud/snow-screened) observations. We then identified all unique Days of Year (DOY, ranging from 1 to 366) for which at least one valid observation existed across the 11-year record. The count of these unique DOYs with available data was then used to define the dynamic harmonic period (T) for that specific pixel in the harmonic model equation. During the model fitting process for these high-latitude pixels, observations falling on DOYs that were identified as "data gaps" (i.e., no valid observations available for that DOY across the entire 11-year record) were excluded. The Julian dates (x) of the remaining, valid observations were then temporally adjusted (e.g., rescaled or shifted based on the sequence of available DOYs) to form a continuous temporal sequence within the model framework. This allowed the harmonic terms of the model to capture the seasonality present in the available data, rather than attempting to force a fit to a 365.25-day cycle that would include large, unobserved periods. This adaptation to a dynamic period significantly improved the stability and accuracy of model fitting in high-latitude regions and dramatically reduced the rate of commission.

Second, computational efficiency was substantially optimized through the implementation of a skipping update strategy for model fitting. To make global processing feasible within a reasonable timeframe, the model was updated only after a larger batch of new observations had been processed since the last successful model fit. The results of this evaluation indicated that updating the model parameters only after accumulating an additional 2% of the available observations since the last model fit provided the optimal trade-off. This strategy maintained a high level of accuracy (the F1-score decreased by less than 0.5% compared to the no-skipping scenario) while reducing the overall computational time by approximately two-thirds.

Finally, an incremental online processing framework was adopted to further enhance efficiency, manage system memory requirements during large-scale production, and enable potential future capabilities for near-real-time updates as new NTL data become available. Instead of processing the entire 11-year time series for each pixel in a single batch operation, which would be highly memory-intensive globally, the change detection was performed incrementally using a fixed-length moving window of input data. As new daily data arrived, it was added to this window, the oldest data potentially dropped off, and the change detection algorithm updated the model and identified changes primarily within this active window. We evaluated the impact of different moving window lengths (ranging from 2 years to 5 years) on the accuracy of change detection compared to processing the full time series. A 4-year moving window was found to provide the optimal balance between accuracy and efficiency, achieving an F1-score (69.05%) very close to that obtained from processing the full time series (F1-score 68.09%) while significantly reducing the instantaneous memory footprint per processing job and allowing for efficient, continuous updates.

$$\hat{\rho}_{(i,x)} = a_i + b_i x + c_i \cos\left(\frac{2\pi}{T} x\right) + d_i \sin\left(\frac{2\pi}{T} x\right) \tag{3}$$

where,

i: The ith observation.

x: Julian date.

$\hat{\rho}_{(i,x)}$: Predicted DNB value for the ith VZA interval at Julian date x.

T: A dynamic period of the number of actual data available day-of-year for each pixel.

a_i: The intercept term for the DNB.

b_i: Coefficient for inter-annual change (trend) for the DNB.

c_i and d_i: Coefficient for intra-annual change for the DNB.

References

1. Li, T. *et al.* Continuous monitoring of nighttime light changes based on daily NASA's Black Marble product suite. *Remote Sens. Environ.* **282**, 113269 (2022).
2. Román, M. O. *et al.* NASA's Black Marble nighttime lights product suite. *Remote Sens. Environ.* **210**, 113–143 (2018).
3. Frey, R. A., Ackerman, S. A., Holz, R. E., Dutcher, S. & Griffith, Z. The Continuity MODIS-VIIRS Cloud Mask. *Remote Sensing 2020, Vol. 12, Page 3334* **12**, 3334 (2020).
4. Cao, C. *et al.* Suomi NPP VIIRS sensor data record verification, validation, and long-term performance monitoring. *Journal of Geophysical Research: Atmospheres* **118**, 11–664 (2013).
5. Cao, C. & Bai, Y. Quantitative Analysis of VIIRS DNB Nightlight Point Source for Light Power Estimation and Stability Monitoring. *Remote Sensing 2014, Vol. 6, Pages 11915-11935* **6**, 11915–11935 (2014).
6. Wang, Z. *et al.* Quantifying uncertainties in nighttime light retrievals from Suomi-NPP and NOAA-20 VIIRS Day/Night Band data. *Remote Sens. Environ.* **263**, 112557 (2021).
7. Wang, Z., Shrestha, R. M., Roman, M. O. & Kalb, V. L. NASA's Black Marble Multiangle Nighttime Lights Temporal Composites. *IEEE Geoscience and Remote Sensing Letters* **19**, (2022).
8. Law, J. Robust statistics—the approach based on influence functions. (1986).
9. Zhu, Z. *et al.* Continuous monitoring of land disturbance based on Landsat time series. *Remote Sens. Environ.* **238**, 111116 (2020).

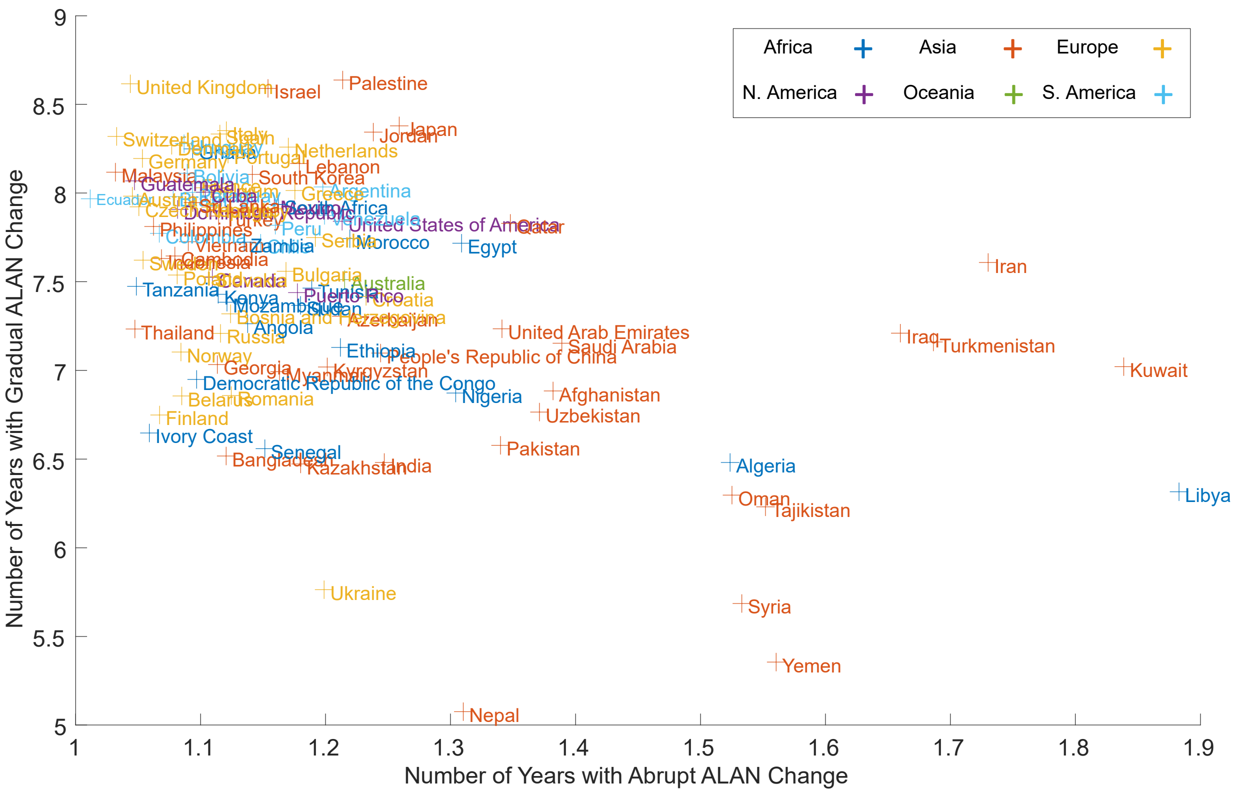

Supplementary Fig. 1 | Abrupt versus gradual ALAN change frequency for the top 100 changing countries/territories (2014-2022). This scatter plot shows the average number of years per 500-meter equal-area sinusoidal changed pixels within countries/territories that experienced abrupt (x-axis) and gradual (y-axis) changes. The top 100 countries/territories are selected based on their total accumulative ALAN change areas from 2014 to 2022. Colors correspond to different continents.

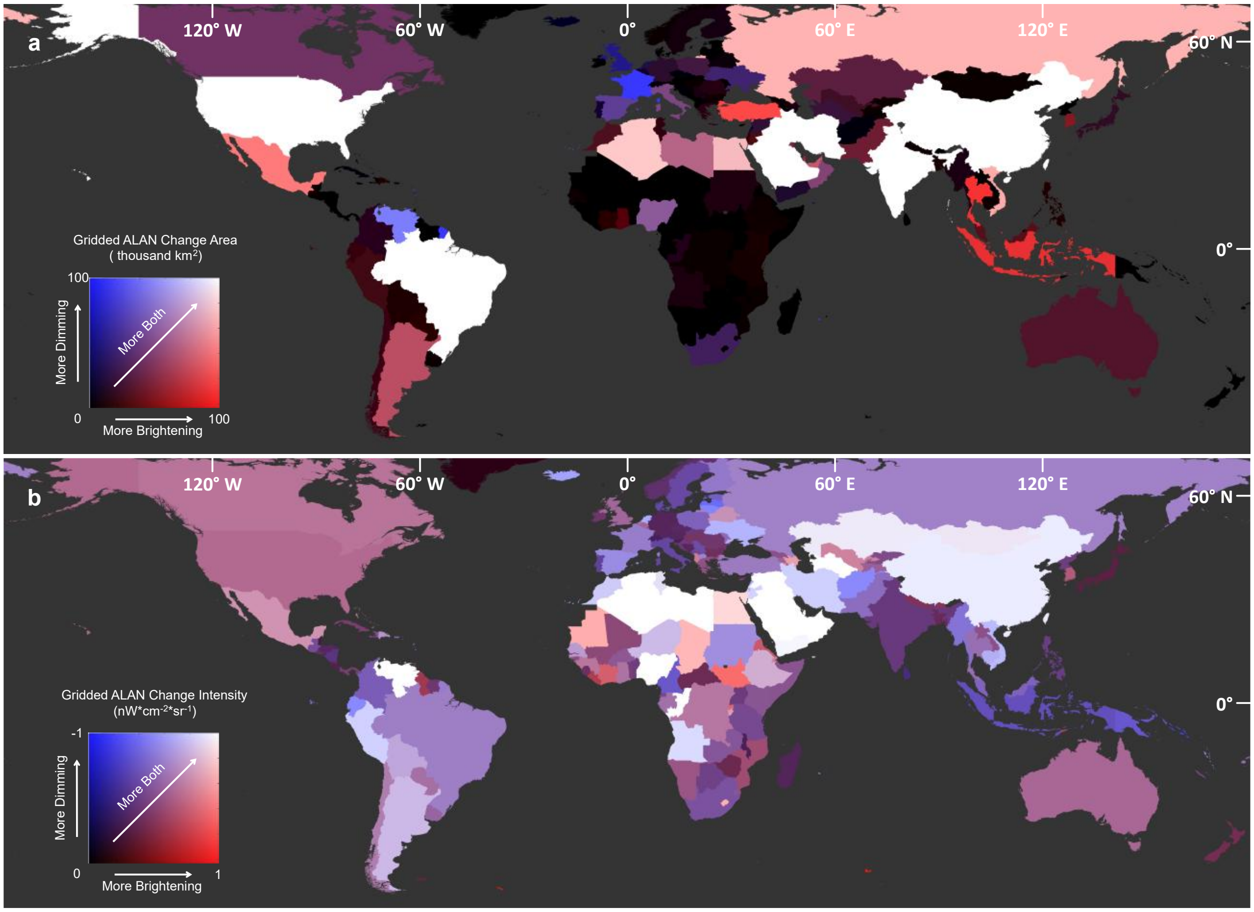

Supplementary Fig. 2 | Country/territory level ALAN changes from 2014 to 2022. a. Total ALAN change area. **b.** The averaged change intensity of detected change. The redder the color, the larger the brightening change. The bluer the color, the larger the dimming change. The whiter colors mean larger changes happened in both brightening and dimming directions.

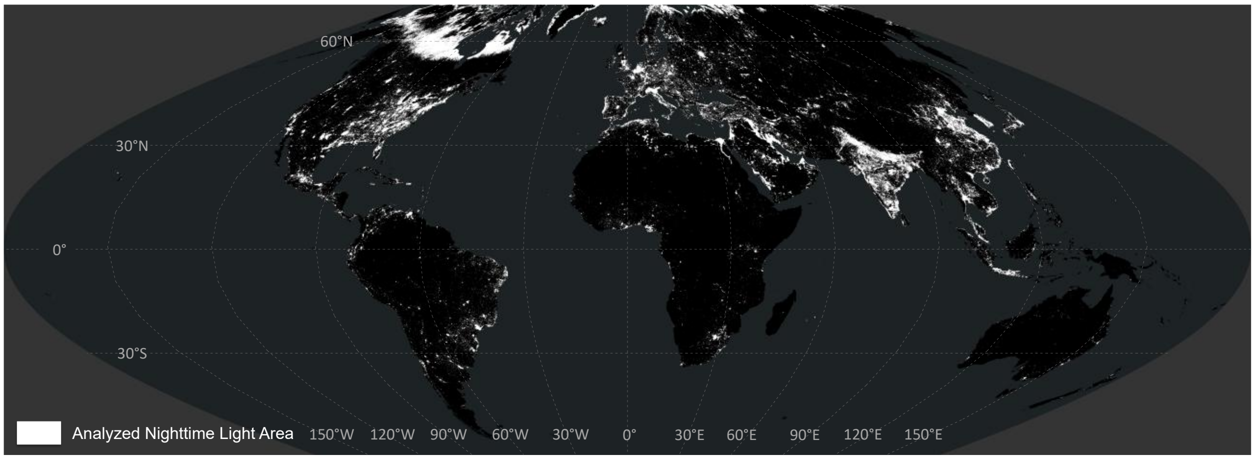

Supplementary Fig. 3 | Analyzed nighttime light area used for global ALAN change detection (70°N-60°S). This map displays the terrestrial areas (white) included in the ALAN change analysis. A pixel was included if its rolling median nighttime light intensity (14 consecutive clear observations) exceeded a 1.0 $nW \cdot cm^{-2} \cdot sr^{-1}$ threshold at any point between 2013 and 2023. Some high-latitude boreal areas appear in this mask (white), which can be due to frequent and intense auroral activity that was persistent enough to pass the initial rolling median filter. We chose not to apply a strict geographic aurora mask in preprocessing to avoid inadvertently excluding potential real, low-level ALAN intensity changes in these regions. However, our VZA-COLD change detection algorithm, which requires sustained, model-breaking trends over 14 consecutive clear observations, is designed to be robust against the typically more transient and variable nature of auroral light. Consequently, these aurora-influenced pixels rarely result in a false positive ALAN change detection, a conclusion supported by the high user's accuracies (low commission errors) of our final change maps. The basemap is the World Continents layer from Esri.

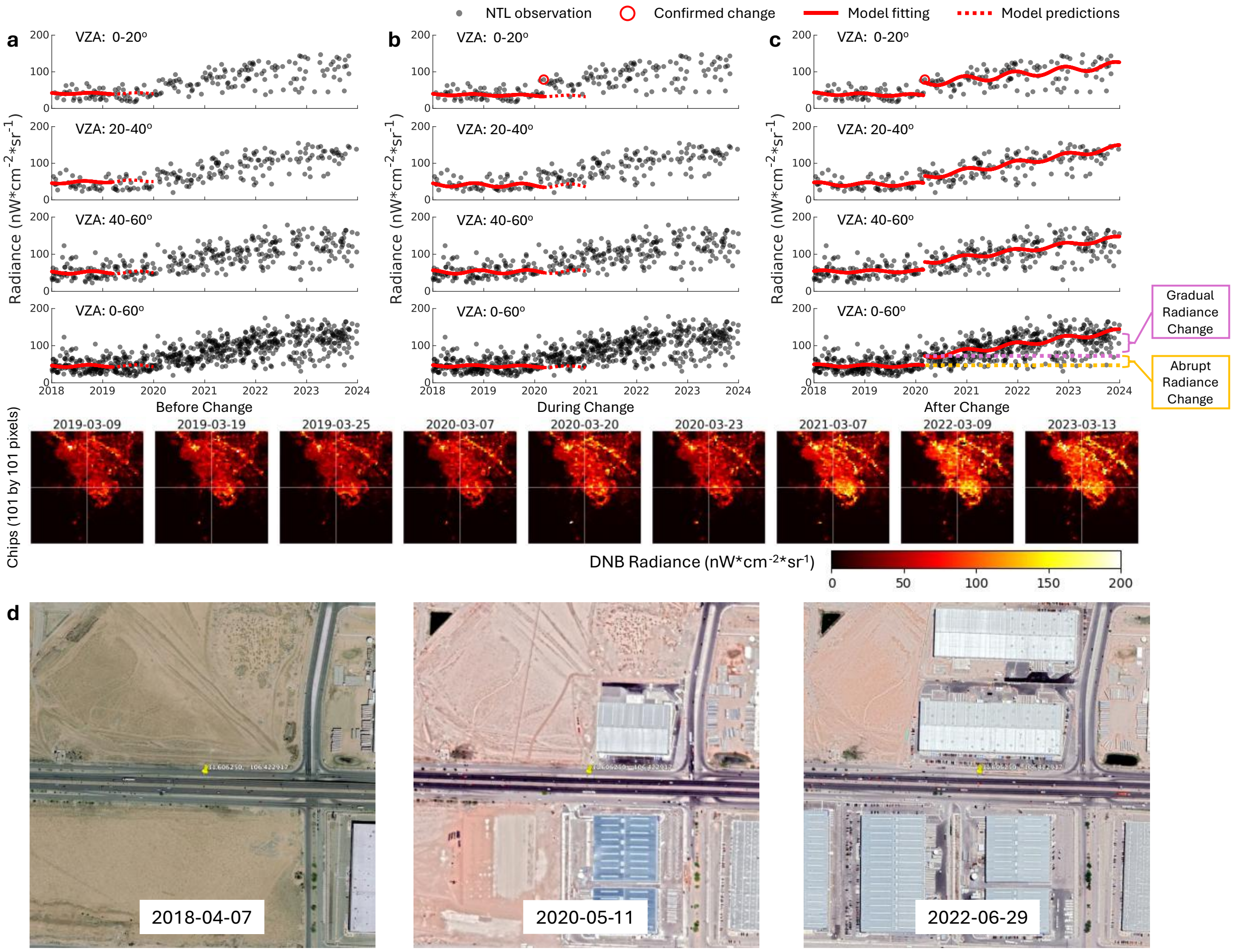

Supplementary Fig. 4 | Illustration of VZA-COLD intermediate change detection progress. Panels **a-c** display the model prediction and change detection before, during, and after the change, respectively. Each panel presents the daily NTL time series for a sample pixel (Lat/Lon: 31.606250, -106.422917; Ciudad Juárez, Mexico) stratified by four Viewing Zenith Angle (VZA) intervals: 0-20° (near-nadir), 20-40°, 40-60° (off-nadir), and 0-60° (all available data). Corresponding daily NTL image chips (101 by 101 pixels; white crosses point to the sample pixel) are displayed below each plot using a consistent color scale for comparison. Panel **d** shows the high-resolution images from Google Earth, providing evidence of new constructions in this area. This location experienced an abrupt change in 2020 followed by a gradual shift. The solid red lines show the estimated time series models, and the dotted lines are the model predicted values. The red circle in Panel **b** is the first break point detected by the VZA-COLD algorithm. Note that due to the large variability of the daily NTL observations from all viewing zenith angles, only the near-nadir interval observations detected the abrupt change, and this break point was used to separate time series models for all stratified intervals. The 0-60° all available data interval time series models are used to calculate the radiance changes from the abrupt and gradual change events (see the 0-60° VZA model in Panel **c**).

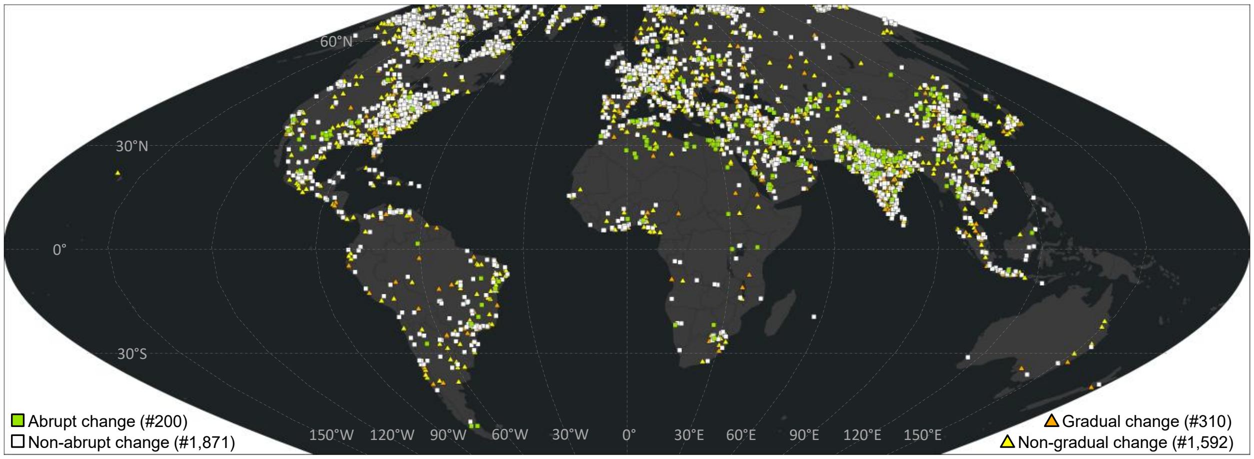

Supplementary Fig. 5 | Spatial distribution of the global validation sample set. The map shows the locations of the n=2,071 abrupt change and n=1,902 gradual change validation sample units used for accuracy assessment, shown on an equal-area sinusoidal projection. The squares show the abrupt ALAN change sample locations, in which the green ones represent the mapped abrupt change samples and the white ones are the mapped non-abrupt change samples. The triangles show the gradual ALAN change sample locations, in which orange shows the mapped gradual change samples, and yellow indicates the mapped non-gradual change samples. The basemap is the World Continents layer from Esri.
